# User Experience and Sustainability of 3D Printing in Dentistry

**DOI:** 10.3390/ijerph19041921

**Published:** 2022-02-09

**Authors:** Tamas Hegedus, Patrik Kreuter, Aron Attila Kismarczi-Antalffy, Tamas Demeter, Dorottya Banyai, Adam Vegh, Zoltan Geczi, Peter Hermann, Michael Payer, Akos Zsembery, Ahmad Al-Hassiny, Khaled Mukaddam, Valentin Herber, Norbert Jakse, Daniel Vegh

**Affiliations:** 1 Department of Prosthodontics, Semmelweis University, Szentkiralyi utca 47., 1088 Budapest, Hungary; hegedus.tamas1@dent.semmelweis-univ.hu (T.H.); gaeczi.zoltan@dent.semmelweis-univ.hu (Z.G.); hermann.peter@dent.semmelweis-univ.hu (P.H.); 2 Faculty of Dentistry, Semmelweis University, Szentkiralyi utca 47., 1088 Budapest, Hungary; kreuterpatrik@gmail.com (P.K.); kismarczi.a.aron@gmail.com (A.A.K.-A.); 3 Department of General Dental Preclinical Practice, Semmelweis University, Szentkiralyi utca 47., 1088 Budapest, Hungary; demeter.tamas@dent.semmelweis-univ.hu; 4 Department of Pediatric Dentistry and Orthodontics, Semmelweis University, Szentkiralyi utca 47., 1088 Budapest, Hungary; banyai.dorottya@dent.semmelweis-univ.hu; 5 Department of Maxillofacial Surgery and Dentistry, Semmelweis University, Maria utca 52., 1088 Budapest, Hungary; vegh.adam@dent.semmelweis-univ.hu; 6 Division of Oral Surgery and Orthodontics, Department of Dental Medicine and Oral Health, School of Dentistry, Medical University Graz, Billrothgasse 4, 8010 Graz, Austria; mi.payer@medunigraz.at (M.P.); valentin.herber@medunigraz.at (V.H.); norbert.jakse@medunigraz.at (N.J.); 7 Department of Oral Biology, Semmelweis University, Nagyvárad tér 4., 1089 Budapest, Hungary; zsembery.akos@dent.semmelweis-univ.hu; 8 Institute of Digital Dentistry, 9 Hillary Court, Lower Hutt, Wellington 5010, New Zealand; director@instituteofdigitaldentistry.com; 9 Department of Oral Surgery, University Center for Dental Medicine Basel (UZB), University of Basel, Mattenstrasse 40, 4058 Basel, Switzerland; khaled.mukaddam@unibas.ch

**Keywords:** 3D printing, social media, dentistry, additive manufacturing, survey, sustainability

## Abstract

Background: 3D printing is a rapidly developing technology in the healthcare industry and in dentistry. Its application clearly shows that this area of digital dentistry has potential for everyday usage across all fields, including prosthodontics, orthodontics, maxillofacial surgery, and oral implantology. However, despite gaining ground, there is a lack of information about how specialists (dentists and dental technicians) use additive technology. Our research group aimed to investigate the impact of social media on additive manufacturing technology among dental specialists and their everyday usage of 3D printing. Methods: This paper investigated specialists’ everyday usage of 3D printers via an online survey (Google Forms). The survey questions aimed to discover the number of 3D printers used, the accessibility of the devices, the annual cost, and the design programs. Since specialists tend to build online communities on social media, we circulated our study questionnaire using our profiles on LinkedIn, Facebook, and Instagram platforms during our research. Results: A total of 120 responses were received from 20 countries, with the most significant numbers being from Hungary 23.7% (*n* = 27), the United States 18.4% (*n* = 21), and the United Kingdom 7.9% (*n* = 9). Most of the participants were dentists (*n* = 68) or dental technicians (*n* = 29), but some CAD/CAM specialists (*n* = 23) also completed our survey. The participants had an average of 3.8 years (±0.7) of experience in the 3D printing field, and owned a total of 405 printing devices (3.6 on average/person). Conclusions: The impact of social media on this research field is growing increasingly. Hence, we support specialists in joining virtual communities on professional platforms. This article intended to provide a practical overview, feedback, and direction for dentists interested in 3D printing technology. From our survey, we can conclude that additive technology is broadening dental applications and the services that we can provide for our patients.

## 1. Introduction

Currently, additive technology is rapidly invading all health care areas, including dentistry [1]. Additive manufacturing (AM), broadly known as 3D printing, transforms how products are designed, produced, and serviced [2]. 3D printing enables creative ideas, performs additive prototyping, makes adjustments to such prototypes, and generally offers flexibility and high performance for industrial and individual customers [3]. As this digital technology has enormous potential and a fast-growing dental field market, knowledge about 3D printing is one of the most significant barriers that can block investment in this technology. However, hardware, software, and exceptional expertise can help us create better and more accurate workflows. Regarding the biomedical usage of AM, it has clearly been shown that this technology is gaining more and more territory in the fields of tumor therapy [4], bone regeneration [5], and bioceramics [6].

3D printing is used in every field of dentistry [7,8], including prosthodontics [9,10,11], orthodontics [12], implantology [13], and maxillofacial surgery [14]. In implantology, the availability of commercially laser-sintered 3D-printed permanent implants is rising [15]. Bone preservation and tissue management plays a huge factor in the success of implant placement; therefore, the new method of 3D-printed scaffolds can help us with oral bone regeneration [16]. Currently, there are many dentists, dental technicians, and CAD/CAM specialists who use additive technology worldwide. However, little knowledge has been collected from the specialists in this technology’s physical usage, and the types of 3D printers used, the number of printers, design software, accessibility of the technology, and annual costs seem to be undiscovered areas. Currently, an increasing number of specialists engaged in 3D printing for dentistry use social media and share their experiences on these platforms.

3D printing is a rapidly developing technology that was introduced over three decades ago [17]. The original disadvantage of this technology was the price, so accessibility was very low for desktop users. The price revolution came in approximately 2010, and one of the core investors in compact 3D printing technology were dentists and dental laboratories. The dental sector was willing to invest, but only if there was a financial advantage. Products need to be standardized and delivered quickly and less expensively over the long term than equivalent products from expensive dental labs. Then, this advantage was far from reality, but these products have brought it closer [18].

Stereolithography Apparatus (SLA) technology has sufficient accuracy such that dentists can use it regularly, and it is currently a core part of digital dentistry [10]. In addition, intraoral scanners have become very popular, as the design and screen can influence patient’s opinion’s, making it easier to sell dental treatment [1,2].

In digital dentistry, 3D printing is a tool that can materialize a product from digital data such as STL or DICOM files [19].

Multiple technologies can be used to create dental products, depending on their additive material and intraoral target use [20], for example, FDM (fused DM), SLA, LFS, DLP, and polyjet. These technologies vary in cost, speed, accuracy, and focus area. For example, FDM printers are the least expensive, while polyjet and DLP are the most costly.

There are several dental applications of 3D printing. For materials, types of metals and resins can be printed. Resin material can be used for models, provisionals, permanent restorations, and removable dentures, among other products. Despite the price difference, it seems apparent that 3D printing technologies offer a better precision and fit to the end product [11].

Digital dentistry is not the future: it is the present. However, it comes at a price, due to the required investment in several types of software and hardware. This technology can be compared with conventional, not digital, methods. However, it has many advantages as well.

Over the past ten years, many dental specialists on social media have encouraged us to recruit participants for our research using different platforms—this also offers clear evidence that social media platforms could be an excellent tool for analysis [21]. As a result, we created social media accounts and groups dedicated to 3D printing in dentistry.

The goal of our social media presence was to collect knowledge and experience in as short a period as possible.

**Figure 1:**
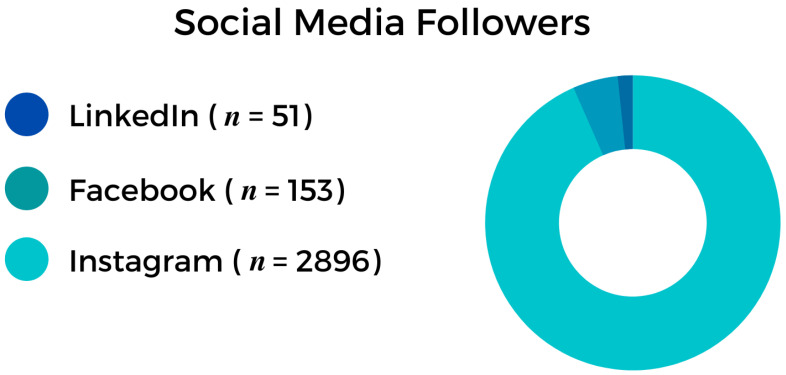
Top social media platforms. We established up profiles on each of these platforms to keep in touch with our followers.

Our leading platform was Instagram with the profile name: @3d_printing_dentistry, but we also have profiles for 3D printing in dentistry on Facebook, Twitter, and LinkedIn (Figure 1). These social media platforms are an excellent way to follow content about various topics, such as dentistry and 3D printing. On social media, dentists showcase reports, techniques and products, or simply advertise their dental office or dental services.

We used social media platforms to create bonds between the curiosity about 3D printing technology and dentistry. In doing so, we provided clear evidence that researchers develop virtual communities to exchange knowledge about their research field [22]. Hence, we believe there is significant potential in those platforms, even though they are not designed to spread scientific information. Additionally, social media’s potential benefits, including greater transparency and access, could help researchers’ gain public trust [23].

## 2. Method

Dentists, dental technicians and CAD-CAM specialists were asked to fill out the questionnaire online. During our research, our aim was that the participants would establish a focus group. It has been clearly shown that focus groups enhance the validity of existing questionnaires by highlighting those concerns held by users and providers that would otherwise have been neglected [24]. People could respond to the questionnaire between 1 January 2020, and 1 January 2021, using a Google Survey form accessible on a computer, tablet, or cell phone.

Participation in the questionnaire survey was voluntary. Exclusion criteria included an incomplete questionnaire or more than one response from the same internet protocol or email address (possible duplicate answers).

People who were not from the industry (non-dental professionals) were excluded from the study. In addition, the questionnaire was in English; only auto-translation to Spanish was available, which might have limited the participation of other language speakers.

### 2.1. Online Survey

We recorded the following information: the first part included 32 questions about the participants and their experience with 3D printing in dentistry.

In contrast, the second part consisted of specific questions about the exact printer model the participant chose to review.

We shared the survey on several social media platforms with direct and indirect content and messages.

Our leading social media platforms were our Instagram page, Facebook, and LinkedIn groups; these functioned as a research community for people experienced in additive technology who use it for their everyday work.

We reviewed the literature to find the most recent information about the sustainability of 3D-printed materials.

### 2.2. Data Collection and Outcome Measures

We collected the data online. All data were stored using Google Survey and Microsoft Excel (Microsoft, Redmond, WA, USA).

### 2.3. Statistical Analysis and Visualization

Data analysis was performed using Prism version 8.4.2. (GraphPad Software Inc., San Diego, CA, USA) software and data are reported as means ± standard deviations (SDs), ranges, or absolute numbers with percentages. We used Pearson’s chi-squared test for statistical analysis. Differences below the 5% limit (*p* < 0.05) were considered significant. For visualization, we used Canva (Canva Pty Ltd., Sydney, Australia).

## 3. Results

We launched our official Instagram page in December 2019 and reached 2944 followers by the end of August 2021. The main page and the post contents share essential information about the digital dental process.

Our followers are primarily specialists in different fields of dentistry, dental students, and dental technicians. We have (as of 22 August 2021) 115 posts and a total of 3786 likes.

Since the beginning of our pilot study, we have been invited by Semmelweis University to present our experience on the impact of social media on 3D printing in dentistry four times:

- Semmelweis University Summer School 2020;
- Semmelweis University Congress for Ph.D. Students 2020;
- Semmelweis University Postgraduate Course—Faculty of Dentistry 2021;
- Semmelweis University Career Day 2021.

We had one Instagram live interview with @3dheals, one of the largest 3D printing communities on Instagram specializing in health care.

We also cooperated with some 3D printing companies (Formlabs and 3D Systems—Nextdent); hence, we were able to organize a product demonstration for the staff at the Semmelweis University Department of Prosthodontics.

Our questionnaire, consisting of 42 questions, received 120 pieces of feedback from 20 countries. Most of these were from Hungary (23.7%, *n* = 27), the United States (18.4%, *n* = 21), and the United Kingdom (7.9%, *n* = 9).

**Figure 2:**
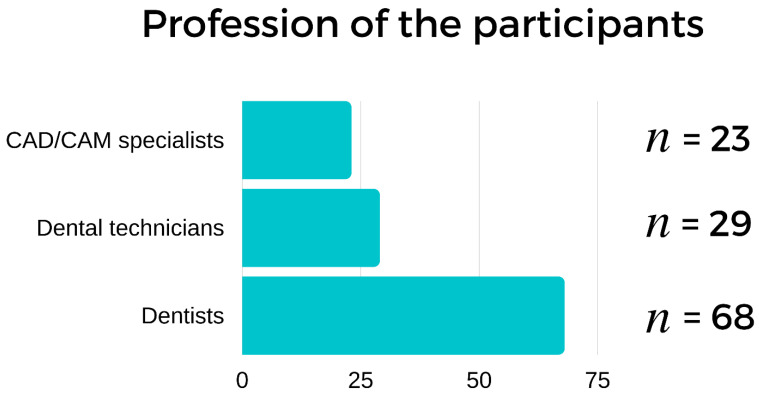
Distribution of participants in terms of profession.

Our feedback came mainly from dentists (*n* = 68), dental technicians (*n* = 29), and CAD/CAM specialists (*n* = 23) (Figure 2.). The participants had an average of 3.8 years (±0.7) of experience in the 3D printing field and owned a total of 405 printing devices (3.6 on average/person).

While most used only one 3D printer, our participants included laboratories with 15–20 devices.

**Figure 3:**
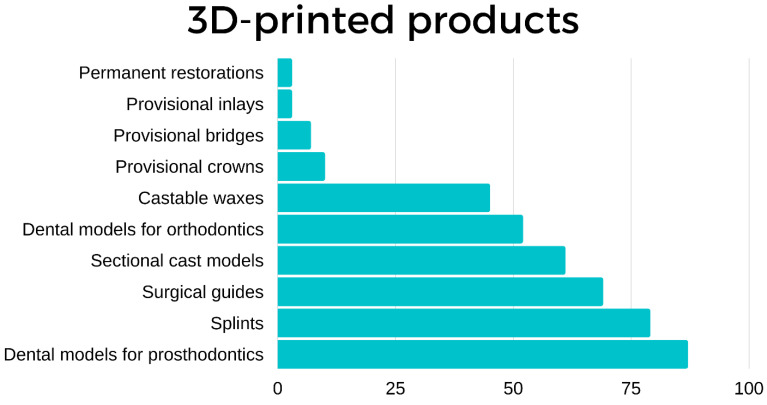
Distribution of the printed products in terms of use.

Regarding their use (Figure 3), most of the respondents commonly used their devices to create dental models for designing prosthodontics (*n* = 87), orthodontics (*n* = 52), sectional cast models (*n* = 61), surgical guides (*n* = 69), castable waxes (*n* = 45), splints (*n* = 79), and provisional restorations (*n* = 17), including dental bridges (*n* = 7), crowns (*n* = 7) and inlays (*n* = 3) and, occasionally, permanent restorations (*n* = 3).

According to 63.3% (*n* = 76) of the respondents, printed models are more accurate than conventional cast models made of gypsum. For the frequency of usage, 72.5% (*n* = 87) of respondents use their device at least every two days, 92.5% (*n* = 111) of them use them at least weekly, and 25.8% (*n* = 31) employ more than 20 L of printing fluid per year.

Most of the participants (55%) (*n* = 66) spent $5000 or less on their printers.

Among the 3D printers used by the participants, the three most popular manufacturers were Formlabs (Formlabs Inc., Somerville, MA, USA) (*n* = 35), Nextdent (Nextdent B.V., Soesterberg, The Netherlands) (*n* = 25), and Asiga (Asiga Inc., Sydney, Australia) (*n* = 24). In addition, several devices from different manufacturers, such as Anycubic (Shenzhen Anycubic Technology Co., Ltd., Shenzhen, China) (*n* = 15), Sprintray (SprintRay Inc., Los Angeles, CA, USA) (*n* = 10), and Phrozen (Phrozen Technology, Hsinchu, Taiwan) (*n* = 11), were used.

When choosing a printer, our respondents usually used three main criteria: accuracy (*n* = 67), printer price (*n* = 64), and recommendations (*n* = 61).

Based on the feedback, we made a comparative evaluation of the three most popular devices among the participants, taking into account the overall satisfaction, the price of the device, the printing material, the printing speed, and the support provided by the manufacturer.

Based on the results (Table 1), users of Asiga (5.0/5.0) and Nextdent (4.9/5.0) printers were the most satisfied with the potential offered by their printers. In terms of price and material, printers manufactured by Anycubic had the highest scores (price: 4.75/5.0, material price: 4.5/5.0). The results showed that the users of NextDent were the most satisfied with the speed of their printers (5.0/5.0).

The responses averaged 4.46 ± 1.3, and only three people expressed dissatisfaction with the printer used.

For the CAD process, the data clearly show that most participants used:

- ExoCad (exocad GmbH, Darmstadt, Germany) (*n* = 41);
- MeshMixer (AutoDesk Inc., San Rafael, CA, USA) (*n* = 29);
- 3Shape (3Shape A/S, Copenhagen, Denmark) (*n* = 21);
- Rayware (SprintRay Inc., Los Angeles, CA, USA) (*n* = 11);
- Chitubox (Shenzhen CBD Technology Co., Ltd., Shenzhen, China) (*n* = 8);
- Blue Sky Plan (Blue Sky Bio, LLC, Libertyville, IL, USA) (*n* = 7);
- Blender (The Blender Foundation, Amsterdam, The Netherlands) (*n* = 1);
- Dental Wings (Dental Wings Inc., Montréal, QC, Canada) (*n* = 1);
- SolidWorks (Dassault Systèmes SolidWorks Corp., Waltham, MA, USA) (*n* = 1) software.

Intraoral scanners (*n* = 73 people) and lab scanners (*n* = 67 people) were used almost the same amount, and often together.

Most respondents preferred 3Shape (3Shape A/S, Copenhagen, Denmark) (*n* = 46) products. Several also indicated that they preferred Medit (Medit Corp., Seoul, Korea) (*n* = 30), Cerec (Dentsply Sirona, Charlotte, NC, USA) (*n* = 17), Itero (Align Technology, Inc., San Jose, CA, USA) (*n* = 13), and Planmeca (Planmeca, OY, Helsinki, Finland) (*n* = 8) products.

Altogether, 55% (*n* = 66) of the respondents became acquainted with the 3D printer they currently use online, and 21.0% (*n* = 24) through their colleagues.

On a 5-point scale, our respondents rated the availability of the devices on average 2.5 points. According to their answers, the delivery time for the printers ranged from immediately up to 2 months.

A total of three people were dissatisfied with the support provided by the manufacturer, and only 51.7% (*n* = 62) of the respondents had received training on the use of the printer by the manufacturer.

## 4. Discussion

The article aimed to provide a practical overview of the feedback and direction for our dentist colleagues willing to invest in this technology. Questionnaires are doubtless one of the primary sources of obtaining data in any research endeavor [25]. It is believed that using different types of procedures for collecting data and obtaining that information through different sources (learners, teachers, program staff, etc.) can augment the validity and reliability of the data and their interpretation [25]. Based on our survey, we can state that additive technology is broadening the applications and services that we can provide for our patients. AM can help support 1-day dentistry, but good preparation is needed before making this investment to ensure a functional and accurate workflow. 3D printing is an excellent tool for materializing idealized forms and restorations, but without perfect knowledge, optimal support, and good services, dentists may face a difficult decision when determining what brand to purchase.

Due to our success in gathering data, we aim to keep our social media community active and provide a space to collect and exchange expertise and knowledge. Furthermore, as new printers, materials, and applications are available every day, 3D printing is an exciting field. Therefore, we aim to investigate various perspectives to benefit both the patient and the dentist.

However, research suggests that printer technology and building orientation heavily affect accuracy [26,27,28,29]. There are existing entry-level 3D printers widespread among young clinicians who are willing to try out this technology. Young professionals belong to the digital-native generation, and investments are more accessible due to low budget requirements (300–500$). However, there is insufficient evidence about their accuracy, except for the study of Lo Giudice et al. [30]. There is a need for further investigations in this manner.

We have suggested recycling materials, such as resin or IPA, as an additional area for obtaining feedback. We found the responses very interesting, and sustainability in 3D printing will be a future target of our research, as we think recycling these materials is crucial to a successful transformation from analog to digital dentistry.

Because we live in a complex ecological crisis of human origin, dealing with sustainability issues, including measuring and mitigating the environmental footprint of technologies related to health care, is inevitable. AM offers a novel method for the versatile, individualized, on-demand production of goods in various fields of the industry, including for dental use, contributing to a future based on intelligent and sustainable manufacturing. However, studies investigating its environmental impact are still scarce, making this topic controversial [31,32].

Electric energy consumption seems to be the highest contributor to the environmental impact of AMs; this is highly dependent on the type of printer, the material used, and the preferences of the manufacturing process [31].

Another environmental concern is the material waste generated from failed prints, byproducts, and supports. The ability to recycle the versatile polymers used in 3DP is limited. These are primarily unrecyclable plastic waste, including the resins used in vat polymerization (SLA), SLS, and material jetting technologies. In powder bed fusion (PBF) technology, the remaining excess metal powder can be collected and reused with nearly 100% efficiency. Moreover, some thermoplastic filaments used in FDM technology can also be reused by reheating. We advocate for establishing systems that enable the return of waste materials to the supplier for recycling, and preventing waste generation by designing objects with minimal support requirements [31,32,33].

3DP processes may give rise to occupational health risks by releasing toxic fumes, volatile organic compounds (VOCs), and ultrafine particles (UFPs) when manufacturing some polymers. The use of fully enclosed 3DP systems and high-efficiency particulate air (HEPA) filters can help to mitigate these concerns [34]. Moreover, prioritizing the development and use of organic polylactic acid (PLA) materials for dental purposes would be advocated [35].

When using SLA technology, uncured resin monomer remnants should be regularly cleaned from the printer and the surface of the printed dental devices with isopropyl alcohol (IPA) as part of the postprocessing protocol [36]. However, excess methacrylate and other uncured resin monomer sediments solved in IPA are dangerous waste, and should be appropriately managed by a waste disposal company. In addition, this poses concerns regarding sustainability and costs, especially in high-capacity 3D printing facilities with increased IPA use. Recycling IPA through distillation and fractionation processes can mitigate these environmental concerns [37].

AM may have numerous beneficial effects on the environment. It offers reduced demand for transportation and thereby reduces carbon footprint, due to the possibility of chairside use and local manufacturing. Moreover, AM offers design-efficient additive production with much lower resource use and material wastage than subtractive manufacturing methods. Apart from several nonrecyclable polymers, 3DP also allows for the versatile usage of eco-friendly thermoplastics such as PLA materials, resulting in biodegradable waste at the end of the product’s life cycle [35]. Dentistry has excellent potential to adapt to new materials for innovative use, and this can hopefully be the case for biodegradable and organic polymers [37]. PLA, for example, could be suitable for use as an eco-friendly provisional restorative material, instead of acrylonitrile butadiene styrene (ABS) [38].

Notably, the spread of AM and 3DP technologies in dentistry may pose a danger of further promoting and strengthening our already widespread societal and cultural misbeliefs regarding consumerism. Moreover, it will neither resolve the fundamental economic problems that support our “throwaway” society, nor curb our pursuit of constant economic growth on a planet with finite biocapacity and resources [34].

Furthermore, to our knowledge, studies investigating the ecological footprint of fully digital workflows in dentistry, including the complete life cycle of AM processes, compared to those of traditional dental laboratory technologies, are unfortunately still missing, choosing between technologies complicated from an environmental point of view.

Finally, we must mention that this study has several limitations: to increase the number of participants, we need to keep growing the social media network we use to reach out to colleagues. Unfortunately, COVID-19 blocked us from participating in live events, workshops and congresses, where the survey advertisement in lectures or abstracts would be much easier. However, we made the first step, and we have chosen social media to test our limits as an independent working group from Semmelweis University. In the future, we need to eliminate those burdens to maintain this data regarding rapidly developing 3D printing technology.

## 5. Conclusions

This survey gave us valuable feedback. 3D printing is one of the essential areas of digital dentistry, and it is expected to spread exponentially. Therefore, dentists need to acquire related knowledge to help them integrate this technology into their everyday practice.

Even though these additive technologies are already widespread, most dentists were not made familiar with 3D printing during their education.

However, the most recent generation of dentists have the appropriate skills and basic knowledge of digital dentistry, and can be quickly introduced to the new workflow offered by 3D printing, which shows their potential for investing in this field.

The renewed digital workflow also takes the patient experience to a new level, allowing new possibilities for representing the expected results via CAD solutions.

The digital process for dental students is not a problem. They learn the critical steps of digital dental treatment during their education, including digital impression taking, intraoral scanning, and additive manufacturing. This new generation of dentists has enormous potential to develop digital dentistry. The working process and patient experience can be created together, as the patient can follow the whole treatment via digital dental tools.

Social media is essential. It is currently having a significant impact on the healthcare industry, and it is also an excellent tool for helping specialists exchange experience and knowledge. In addition, if one need’s help, it seems to be a faster method for receiving it than the official support system.

This pilot study aimed to develop digital dental treatment and plan investments at Semmelweis University based on the participants’ responses. In addition, the research group’s focus was to build a community and provide help for dentists and dental students interested in additive manufacturing.

## Figures and Tables

**Table 1:** Comparison of different types of 3D printers used for dental purposes (bold: highest value in the column. The participant’s general satisfaction level was measured on a 5-grade scale.

Company	Satisfaction	Price	Material Price	Speed	Support	Overall
Asiga	**5.0** (*n* = 24)	3.9 (*n* = 24)	3.7 (*n* = 24)	4.4 (*n* = 24)	**4.7** (*n* = 24)	4.3 (*n* = 24)
Nextdent	4.9 (*n* = 25)	4.4 (*n* = 25)	4.4 (*n* = 25)	**5.0** (*n* = 25)	4.6 (*n* = 25)	**4.7** (*n* = 25)
Formlabs	4.4 (*n* = 35)	4.4(*n* = 35)	4.3 (*n* = 35)	2.6 (*n* = 35)	4.2 (*n* = 35)	4.0 (*n* = 35)
Anycubic	4.5 (*n* = 15)	4.8 (*n* = 15)	**4.5** (*n* = 15)	4.0 (*n* = 15)	4.3 (*n* = 15)	4.4 (*n* = 15)
Sprintray	4.3 (*n* = 10)	4.3 (*n* = 10)	4.3 (*n* = 10)	4.0 (*n* = 10)	4.0 (*n* = 10)	4.2 (*n* = 10)
Phrozen	3.7 (*n* = 11)	**5.0** (*n* = 11)	4.0 (*n* = 11)	4.0 (*n* = 11)	3.0 (*n* = 11)	3.9 (*n* = 11)

## Data Availability

The datasets used and/or analyzed during the current study are available from the corresponding author on reasonable request.

## Abbreviations

3D	3 Dimensional
3DP	3-Dimensional Printing
ABS	Acrylonitrile Butadiene Styrene
AM	Additive Manufacturing
CAD/CAM	Computer-Aided Design/Computer-Aided Manufacturing
DiCOM	Digital Imaging and Communications in Medicine
DLP	Digital Light Processing
FDM	Fused Deposition Modeling
HEPA	High-Efficiency Particulate Air
IPA	Isopropyl Alcohol
LFS	Low Force Stereolithography
PBF	Powder Bed Fusion
SLA	Stereolithography Apparatus
STL	Standard Triangle Language
UFP	Ultra Fine Particles
VOC	Volatile Organic Compounds
